# Electrocardiographic and enzymatic correlations with outcome in neonates with hypoxic-ischemic encephalopathy

**DOI:** 10.1186/1824-7288-38-33

**Published:** 2012-07-23

**Authors:** Jyoti Agrawal, Gauri S Shah, Prakash Poudel, Nirmal Baral, Ajay Agrawal, Om P Mishra

**Affiliations:** 1 Departments of Pediatrics and Adolescent Medicine, B.P. Koirala Institute of Health Sciences, Dharan, Nepal; 2 Departments of Biochemistry, B.P. Koirala Institute of Health Sciences, Dharan, Nepal; 3 Departments of Obstetrics and Gynecology, B.P. Koirala Institute of Health Sciences, Dharan, Nepal; 4 Department of Pediatrics, Institute of Medical Sciences, Banaras Hindu University, Varanasi, India

**Keywords:** Perinatal asphyxia, HIE, Myocardial dysfunction

## Abstract

**Background:**

Perinatal asphyxia leading to hypoxic-ischemic encephalopathy (HIE) is a common problem causing multi organ dysfunction including myocardial involvement which can affect the outcome.

**Objective:**

To evaluate the myocardial dysfunction in neonates having HIE by electrocardiographic(ECG) and cardiac enzymes (CK Total, CK-MB and Troponin I) and find out the relationship with HIE and outcome.

**Design/Methods:**

This was a hospital based prospective study. Sixty term neonates who had suffered perinatal asphyxia and developed HIE were enrolled. Myocardial involvement was assessed by clinical, ECG, and CK Total, CK-MB and Troponin I measurements.

**Results:**

Of 60 cases, 13(21.7%) were in mild, 27(45%) in moderate and 20(33.3%) belonged to severe,HIE. ECG was abnormal in 46 (76.7%); of these 19 (41.3%) had grade I, 13 (28.2%) grades II and III each and 1 (2.1%) with grade IV changes. Serum levels of CK Total, CK- MB and Troponin I were raised in 54 (90%), 52 (86.6%) and 48 (80%) neonates, respectively. ECG changes and enzymatic levels showed increasing abnormalities with severity of HIE, and the differences among different grades were significant (p = 0.002, 0.02, <0.001 and 0.004, respectively). Nineteen (32%) cases died during hospital stay. The non- survivors had high proportion of abnormal ECG (p = 0.024), raised levels of CK-MB (p = 0.018) and Troponin I (p = 0.008) in comparison to survivors.

**Conclusions:**

Abnormal ECG and cardiac enzymes levels are found in HIE and can lead to poor outcome due to myocardial damage Early detection can help in better management and survival of these neonates.

## Introduction

Perinatal asphyxia is a common problem with the incidence varying from 0.5 – 2% of live births [1,2]. It accounts for 9.7/1000 live births and contributes for 30% of neonatal mortality in Nepal [3]. It is an important cause of admission to neonatal intensive care units (NICU) with multi organ dysfunction [4]. When an asphyxic event occurs, it leads to a series of physiological mechanisms in order to preserve the function of vital organs (brain and heart), whereas other organs such as the kidneys, gastrointestinal tract, and skin are affected to a varying degree based on the duration of the episode [5,6]. However, in spite of compensatory mechanisms, it may progress to hypoxic- ischemic encephalopathy (HIE) involving the brain and heart [7].

The incidence of cardiac dysfunction in perinatal asphyxia varies from 24–60% [1]. Apart from the clinical presentation, electrocardiography (ECG), echocardiogram and determination of cardiac enzymes are useful tools to detect myocardial involvement. In contrast to adults, recognition of myocardial ischemia is far more difficult in neonates. Only few studies have assessed the myocardial dysfunction with assay of cardiac enzymes and ECG abnormalities [8,9].

Rajakumar et al. [10] demonstrated that C-Troponin assay is useful in evaluating the severity of myocardial damage and outcome in perinatal asphyxia. Kanik et al. [11] studied the assessment of myocardial dysfunction in neonates with HIE to predict the mortality. However, there is paucity of reports from our country where mothers often report to hospital quite late with obstetric complications and neonates suffer from birth asphyxia with high mortality. So it becomes imperative to detect myocardial dysfunction as one of the contributing factor and to provide early treatment. Therefore, the present study was undertaken to find out the myocardial involvement in neonates having HIE by the ECG and cardiac enzymes (CK-MB, Troponin I) changes and its correlation with various degree of HIE and outcome.

## Patients and methods

This was a prospective study conducted at a tertiary care centre of a teaching hospital during the period of February, 2010 to January, 2011. The protocol of the study was approved by the Institute Ethics Committee and written informed consent for inclusion was obtained from the parents of each neonate. Sixty inborn neonates at term gestation, who suffered from perinatal asphyxia and developed to hypoxic ischemic encephalopathy, as defined by Levene staging [12], were included. APGAR score of newborns was evaluated at 1, 5 and 10 minutes and they were resuscitated as per NRP guidelines [13]. Cases with congenital heart diseases, major central nervous system malformations and neonatal sepsis were excluded. All the neonates were managed in NICU as per hospital protocol. They were given oxygen by hood (5–6 l/min), nasal continuous positive airway pressure, mechanical ventilation (based on saturation of oxygen (SpO2) and Arterial Blood Gas findings), intravenous fluids, vitamin K, inotropes (Dopamine and/or Dobutamine each by 1–20 μg/kg/min) and anticonvulsants (Phenobarbitone 20 mg/kg as loading dose, followed by 3–5 mg/kg/day, and phenytoin sodium was also added with same dose in non-responder to phenobarbitone),wherever required. First line antibiotics (cefotaxime and gentamycin) were given to those cases where risk factors for sepsis were present and required mechanical ventilation. Feeding was started once patient showed improvement, initially started as nasogastric feeding and then followed by spoon or breast feeding.

A 12-lead ECG was recorded and 4 ml of venous blood was collected for cardiac enzymes estimation in each asphyxiated neonates within 72 hours of life. Infants with ECG changes of grade 1 or 2 were diagnosed to have mild, whereas those with changes of grades 3 or 4 were considered to have severe injuries. The grading was done as per criteria defined by Jedeikin et al. [14].

Grade1 - with flat or inverted T waves on 1 or 2 leads except AVR.

Grad 2- with flat or inverted T-waves in 3 or more leads except AVR.

Grade 3- with flat or inverted T-waves in 3 or more leads and either ST depression or elevation >2 mm in at least two chest leads or >1 mm in at least two standard leads, or a Q-wave abnormality of duration >0.02 s or amplitude >25% of R wave in one anterior or three related chest leads.

Grad 4- presence of classical segmental infarction with abnormal Q-wave and markedly elevated ST segment or complete left bundle branch block.

Creatine kinase-Total (CK-Total), Creatine kinase-MB (CK-MB) were measured by quantitative determination based on immune-inhibition IFCC methodology (Agappe diagnostics limited) using semi auto-analyzer. The Calbiotech cTnI ELISA was used for the quantitative determination of cardiac Troponin I from samples. The cTnI ELISA is based on the principal of a solid phase ELISA. . Specimen which could not be assayed within 24 hrs was frozen at −20 °C and was stable for 6 months.

A chest X-ray was taken in all patients. The serum which was collected from patient were also analysed for other hematological and biochemical investigations like PCV, total and differential leukocyte count, urea, creatinine, sodium, potassium, calcium and blood sugar.

### Statistical analysis

Differences in mean of quantitative data among different stages of HIE were compared by Analysis of Variance (ANOVA) and proportions by Chi-Square tests. Median values of CK total, CK-MB and troponin I were compared by Kruskal- Wallis test and post-hoc analysis was done to find out the difference between the two parameters. A p value of < 0.05 was considered as statistically significant.

## Results

The basic characteristics of neonates according to various HIE stages are presented in Table 1. It was observed that none of the parameters showed significant difference among different stages of HIE. The HIE staging in relation to various natal factors is shown in Table 2, and it showed that color of liquor and mode of delivery did not affect the development of different stages of HIE. However, modes of resuscitation and Apgar scores at 1, 5 and 10 minutes were statistically significant among different stages of HIE. Seventeen (28.3%) neonates required initial steps of resuscitation and in 43 (71.6%) bag and mask ventilation was given.It was switched over to bag and tube ventilation in 6 cases as they required prolonged ventilation, in addition 4 (6.6%) needed chest compression also.

**Table 1:** Basic characteristics of neonates and HIE staging

**Parameters**	**Categories**	**HIE Stage**			**p**
		**Mild (n = 13)**	**Moderate (n = 27)**	**Severe (n = 20)**	
Gender	Male	11 (84%)	21 (77%)	17 (85%)	0.780*
	Female	2 (16%)	6 (23%)	3 (15%)	
Registration Status	Booked	0 (0%)	1 (3.7%)	1 (5%)	0.729*
	Registered	1 (7.6%)	1 (3.7%)	0 (0%)	
	Unbooked	12 (92.4%)	25 (92%)	19 (95%)	
Gestational age	Term	8 (62%)	23 (85%)	16 (80%)	0.230*
	Post dated	5 (38%)	4 (15%)	4 (20%)	
Antenatal complications	No	12 (92%)	18 (67%)	14 (70%)	0.210*
	Yes	1 (8.0%)	9 (33%)	6 (30%)	
Obstetric Problems	No	13 (100%)	25 (92%)	20 (100%)	0.282*
	Yes	0 (0%)	2 (7.4%)	0 (0%)	
Birth weight (g)		2826.92 ± 446.1	3111.11 ± 437.94	2875 ± 331.46	0.06**
Length (cm)		49.58 ± 2.02	50.3 ± 3.05	49 ± 2.2	0.241**
Head circumference (cm)		34.31 ± 1.49	34.07 ± 1.64	33.75 ± 1.25	0.558**
Maternal age (yr)		22.62 ± 3.01	24.15 ± 3.98	23.45 ± 4.55	0.521**
Maternal weight (kg)		58 ± 8.11	56.07 ± 5.92	58.0 ± 8.4	0.587**

n = Number of cases,*Chi-square test, ** ANOVA.

**Table 2:** HIE staging in relation to natal factors

**Birth Details**	**Categories**	**HIE Stage**			**P**
		**Mild (n = 13)**	**Moderate (n = 27)**	**Severe (n = 20)**	
Liquor	Clear	3 (23%)	17 (63%)	10 (50%)	0.061*
	Meconium	10 (77%)	10 (37%)	10 (50%)	
Modes of delivery	Normal	10 (77%)	19 (70.4%)	14 (70%)	0.900*
	Caesarean	3 (23%)	6 (22.2%)	5 (25%)	
	Instrumental	0 (0%)	2 (7.4%)	1 (5%)	
Details of resuscitation	Initial steps	3 (23%)	13 (48%)	1 (5%)	0.009*
	Bag and Mask ≠ Chest compression	10 (77%)	14 (52%)	19 (95%)	
		0 (0%)	0 (0%)	4≠	
Apgar score-1 min		3.23 ± 0.83	3.11 ± 0.64	2.55 ± 0.6	0.007**
Apgar score-5 min		5.08 ± 0.49	4.78 ± 0.58	4.1 ± 0.79	<0.001**
Apgar score-10 min		7.46 ± 0.88	6.96 ± 0.9	5.5 ± 1.15	<0.001**

≠ In 6 neonates, bag and mask was switched over to bag and tube ventilation and 4 cases required chest compression also n = Number of cases, * Chi-square test, ** ANOVA.

**Figure 1:**
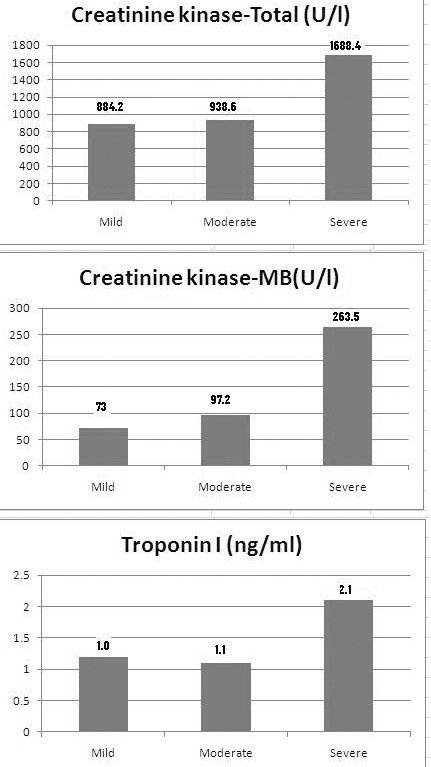
Bar diagram showing median values of CK Total, CK-MB and Troponin I in different stages of HIE.

The abnormalities in X-ray chest, ECG and cardiac enzyme are presented in Table 3. The chest X-ray was abnormal and showed features of meconium aspiration in 11 (18.3%) cases. ECG changes were observed in 46 (76.7%) neonates; of these 19 (41.3%) had grade I, 13 (28.2%) grades II and III each and 1 (2.1%) grade IV abnormalities. Six neonates with mild HIE while 20 each in moderate and severe HIE had abnormal ECG and changes among different stages of HIE were significant (P = 0.002). The cardiac enzymes levels were considered abnormal when it were above our reference laboratory values (CK Total > 190 U/l, CK-MB >25 U/l and troponin I > 0.05 ng/ml). The serum level of enzyme CK Total was raised in 54 (90%), while CK- MB was raised in 52 (86.6%) neonates with marked elevation (> 100 U/L) in 34(56.6%). The troponin I was also raised in 48 (80%) cases. The mean levels of CK Total, CK-MB and troponin I were 1176.8 U/l, 147.5U/l and 1.4 ng/ml, respectively. Their levels showed further rise with increasing severity of HIE and the severe HIE group had significantly higher level in comparison to mild and moderate HIE cases (P = 0.02, <0.001 and 0.004, respectively). (Figure 1)

**Table 3:** X-ray chest, ECG and cardiac enzymes in study subjects

**Investigations**	**Categories**	**Number of patients**	**Percentage**
Chest X-ray	Normal	49	81.7
	Abnormal	11	18.3
E.C.G	Abnormal	46	76.7
	Normal	14	23.3
Grading of E.C.G	Grade1	19	41.3
	Grade2	13	28.2
	Grade3	13	28.2
	Grade4	1	2.1
CK Total (U/l)	<190	6	10
	>190	54	90
Mean ± SD of CK Total		1176.8 ± 1023.1	
CK MB (U/l)	<25	8	13.3
	25-100	18	30
	>100	34	56.6
Mean ± SD of CK MB		147.5 ± 161.0	
Troponin I (ng/ml)	≤0.5	12	20
	>0.5	48	80
Mean ± SD of Troponin I		1.4 ± 1.1	

Three babies (1 mild and 2 moderate HIE) left against medical advice because their parents had financial constraints in continuing the medication in hospital. Sixteen of 57 (28%) neonates died during hospital stay between 30 to 264 hr of post natal life. They belonged to severe HIE stage and needed mechanical ventilation, inotropes and anticonvulsants in addition to other supportive measures. Outcome of newborns with HIE in relation to ECG, CK-Total, CK-MB and troponin I are shown in Table 4. The non- survivors had significantly high proportion of abnormal ECG (p = 0.01), raised levels of CK-MB (p = 0.03) and troponin I (p = 0.01) in comparison to survivors. However, no such relationship was observed with CK-Total level in these neonates.

**Table 4:** Outcome of neonates with HIE in relation to ECG and cardiac enzymes

**Parameters**	**Categories**	**Survivors (n = 41)**	**Non-survivors (n = 16)**	P*
ECG	Normal	13 (31.7%)	0 (0%)	0.010
	Abnormal	28 (68.3%)	16 (100%)	
CK-Total(U/L)	Normal	4 (9.8%)	1 (6.3%)	0.674
	Abnormal	37 (90.2%)	15 (93.8%)	
CK-MB(U/L)	Normal	10 (24.4%)	0 (0%)	0.030
	Abnormal	31 (75.6%)	16 (100%)	
Troponin-I (ng/dl)	Normal	12 (29.3%)	0 (0%)	0.015
	Abnormal	29 (70.7%)	16 (100%)	

*Chi-square test.

## Discussion

As such various demographic data such as gender, birth weight, length, head circumference, maternal registration status, gestational age, antenatal complications, obstetric problems, maternal age and weight were evenly distributed among different stages of HIE.

Meconium stained amniotic fluid was present in 50% of cases which is similar to the finding of Martin-Ancel et al. [15]. Modes of delivery and meconium staining of liquor did not affect the progression to different stages of HIE in neonates with birth asphyxia.

Apgar score at 1 minute was <3 in 14(23.3%) and 3–5 in 46(76.7%) neonates. Apgar score at 5 minute was 3 to 5 in 57(95%) and >5 in 3(5%) cases. In a study by Shah et al. [4], Apgar score at 5 minute was less than 5 in (63.12%) neonates. Martin-Ancel et al. [15] also reported Apgar score at 1 minute was 4 in 70% of cases which is similar to our study. However, Apgar scores at 5 minute were ≥7 in 46%, 4 to 6 in 37%, and ≤ 3 in 14% of patients which is in contrast to our findings. In our study, 41(68.3%) neonates had seizures. Shah et al^4^ documented seizures or coma in 87% of cases.

The various clinical features related to cardiac dysfunction documented were respiratory distress, congestive cardiac failure and shock. We found respiratory distress in 32(53.3%) and shock in 29(48.3%) cases. One neonate had congestive cardiac failure which was present in HIE stage III group. Rajakumar et al. [10]*, reported* respiratory distress in 20 (66.7%), cardiac failure in 11 (36.7%) and cardiogenic shock in 5 (16.7%); while 8 babies were asymptomatic. However, Mandal et al. [16] found congestive cardiac failure in higher percentage (36%) of their cases. They found shock in 44%, which is similar to our observation.

Pulmonary infiltrates suggestive of meconium aspiration were present in X-ray chest of 11(18.3%) neonates and of these, 9 cases belonged to severe HIE. ECG changes were present in 46(76.7%) neonates. Grade 1 ECG changes were seen in 19(41.30%), grades 2 and 3. in 13(28.26%) cases each. Rajakumar et al. [10] reported ECG changes in almost similar percentage (73.3%) of cases. The most common finding in their study was ‘T’ wave inversion (36.7%) followed by ‘T’ wave flattening (33.3%), which is equivalent, to grade 1 ECG change. These ECG abnormalities indicate myocardial ischaemia due to birth asphyxia in neonates.

Mean CK total level, CK MB and troponin I were 1176.8 IU/l, 147.5 IU/l and 1.4 ng/ml, respectively. Mandal et al. [16] and Warburton et al [17] reported CK-MB values as high as a 823.5 IU/l and 328 IU/l, respectively. Rajakumar et al [10] reported lower mean CK-MB level (121 IU/l) in their study. The enzyme levels showed significant rise with increasing severity of HIE; indicating more myocardial ischaemia in severe HIE than mild and moderate cases.

Primhak et al [18] studied serial electrocardiogram and CK-MB in term infants and found that CK-MB was associated with myocardial injury in asphyxiated infants. However, Omokhodion et al [19] concluded that specificity of CK-MB as a marker of myocardial injury in asphyxiated newborns is possible but remains uncertain. Borke and co-workers [20] showed cardiac troponin I to be a reliable marker of myocardial necrosis. Thus previous studies showed that perinatal asphyxia is associated with myocardial dysfunction. However, they did not provide information regarding interventions performed in the delivery room and moreover study was conducted prior to the introduction of standardized neonatal resuscitation [13].^.^

The influence of hypoxemia related myocardial dysfunction in the newborn occurs in 30% of asphyxiated neonetes [15] and is thought to be secondary to direct effect of ischaemia on cardiac myocytes. Enzymatic indicators of myocardial injury in the asphyxiated newborns are elevated and often associated with electrocardiographic changes. The measurement of cardiac troponin I may have a role in the early identification of neonates with myocardial damage secondary to ischaemia. However, cardiac abnormalities often are under diagnosed and require a high index of suspicion.

We found a clear relationship between outcome of asphyxiated newborns and alterations in ECG and enzymatic parameters as significantly higher proportions of non- survivors had ECG abnormalities and raised levels of CK-MB and troponin I. Patients showing more severe hypoxic damage also reflected the striking changes in these parameters. Hypoxia is responsible for multiorgan dysfunction causing deaths, but particularly results in myocardial damage despite ``preferential'' myocardial perfusion [5,21]. These markers may be useful in providing appropriate cardiovascular support in addition to managing the primary condition.

Thus, it can be concluded that myocardial dysfunction secondary to perinatal asphyxia is more frequent than thought, for which it will be useful to submit asphyxiated neonates to ECG monitoring and assay of cardiac enzyme markers complemented with clinical findings. The early detection and prompt treatment of condition will help in improving prognosis of these asphyxiated newborns.

## Conclusions

This study suggests that significant ECG and cardiac enzymes (CK-total, CK-MB, troponin I) alterations occur in neonates with birth asphyxia having HIE. These features indicate hypoxic damage to myocardium affecting the survival.

### What is already known on this topic

Varying degree of multi organ dysfunction including myocardial involvement in perinatal asphyxia developing HIE can occur.

### What this study adds

Different grades of ECG and cardiac enzymes abnormalities develop in neonates with HIE and it adversely affect the outcome.

## Competing interests

The authors declare that they have no competing interests.

## Authors’ contributions

GSS & PP- involved in concept and design of the study, JA & AA- collection and analysis of data, NB- helped in biochemical analysis and OPM- analysis of data and drafting the manuscript. All authors read and approved the final manuscript.
